# Cost of Dry Eye Treatment in an Asian Clinic Setting

**DOI:** 10.1371/journal.pone.0037711

**Published:** 2012-06-11

**Authors:** Samanthila Waduthantri, Siew Sian Yong, Chien Hua Tan, Liang Shen, Man Xin Lee, Sangeetha Nagarajan, Mynt Htoon Hla, Louis Tong

**Affiliations:** 1 Singapore Eye Research Institute, Singapore, Singapore; 2 Singapore National Eye Centre, Singapore, Singapore; 3 Duke-NUS Graduate Medical School, Singapore, Singapore; 4 National University of Singapore, Singapore, Singapore; University of Missouri-Columbia, United States of America

## Abstract

**Objectives:**

To estimate the cost and patterns of expenditure of dry eye treatment.

**Methodology:**

We retrieved data on the type and cost of dry eye treatment in Singapore National Eye Centre from pharmacy and clinic inventory databases over a 2 year period (2008–2009) retrospectively. According to the type of treatment, data were sorted into 7 groups; meibomien gland disease (MGD) treatment, preservative free lubricant eye drops, preserved lubricant eye drops, lubricant ointments and gels, cyclosporine eye drops, oral supplements and non-pharmacological treatments/procedures. Each recorded entry was considered as one patient episode (PE). Comparisons in each group between two years were carried out using Pearson Chi-Square test. Significance level was set at alpha  = 0.05.

**Results:**

Cost data from 54,052 patients were available for analysis. Total number of recorded PEs was 132,758. Total annual expenditure on dry eye treatment for year 2008 and 2009 were US$1,509,372.20 and US$1,520,797.80 respectively. Total expenditure per PE in year 2008 and 2009 were US$22.11 and US$23.59 respectively. From 2008 to 2009, there was a 0.8% increase in total annual expenditure and 6.69% increase in expenditure per PE. Pharmacological treatment attributes to 99.2% of the total expenditure with lubricants accounting for 79.3% of the total pharmacological treatment expenditure. Total number of units purchased in preservative free lubricants, cyclosporine eye drops and MGD therapy have increased significantly (p<0.001) whereas number of units purchased in preserved lubricants and ointments/gels have reduced significantly (p<0.001) from 2008 to 2009.

**Conclusion:**

Dry Eye imposes a significant direct burden to health care expenditure even without considering indirect costs. Health care planners should be aware that these direct costs appear to increase over the time and more so for particular types of medications. Given the limitations of socio-economic data, true societal costs of Dry eye syndrome are likely to be much higher than estimated.

## Introduction

Dry eye syndrome (DES) is a multifactorial chronic disease that affects millions of people over the world with significant socio-economic implications, including expenses associated with increased health care utilization (e.g. medication and physician visits) and impact on daily social and physical functioning, work place productivity and quality-of-life [1]–[4].

Traditional treatment for DES has been largely palliative with over the counter lubricating eye drops or artificial tears. Increase in knowledge of patho- physiology of DES has led to tremendous advances in treatment in the last two decades. Lately, with introduction of newer modalities such as topical cyclosporine emulsion [2], [5], [6], mucomimetic drugs and oral supplements [2], [7] which have substantial acquisition price, the burden of patient’s pharmacy budget has significantly increased6. Extensive cost of dry eye treatment may affect compliance to treatment, the choice of medication by clinicians, stocking of medication in hospitals, and the possibility of medications to be listed in standard formulary.

Evaluating cost effectiveness of dry eye treatment is quite challenging due to the multifactorial nature of the disease and potential limitations of techniques available to evaluate therapeutic outcomes of multi-palliative treatment modalities used. In US cost of managing dry eye patients in health care organizations is estimated at US$700,000 per million patients [8]. There are large variations in the dry eye treatment costs between countries in Europe [9]. The absolute costs may be much higher for the Asian population as the prevalence rates of dry eye in Asian population is higher (30%) compared to predominant Caucasian populations (15%) [8].

There is lack of published data on healthcare resource utilization in managing DES in Asian countries. In this study we report the cost and patterns of expenditure of dry eye treatment in a Singapore population.

## Materials and Methods

### Study Design

This is a retrospective cost analysis study.

### Methods

We retrieved the data retrospectively on type and cost of dry eye treatment prescribed by ophthalmologists in Singapore National Eye Centre (SNEC) from pharmacy and clinic inventory databases over a 2 year period (2008–2009). SNEC is the designated national centre for tertiary eye care services in Singapore which currently manages an annual workload of 250,000 outpatient visits, which amounts to around 60% of the overall eye care in the public sector. (Available: http://www.snec.com.sg/about/history/Pages/home.aspx, http://www.snec.com.sg/about/achievements/Pages/home.aspx).

We included cost data on dry eye treatment of all the patients that attended the outpatient eye clinics in SNEC from year 2008 to 2009 in our analysis. Data on prescription drugs purchased elsewhere other than SNEC pharmacy were not captured.

According to the type of treatment, data were sorted into 7 groups; Group A: Treatment for meibomian gland disease (MGD), Group B: Preservative free lubricant eye drops, Group C: preserved lubricant eye drops, Group D: lubricant ointments and gels, Group E: Cyclosporine eye drops, Group F: Oral supplements Group G: Non pharmacological treatments/procedures.

Treatment for MGD included warm compress with eye masks and lid hygiene with Blephagel® (Spectrum Thea Pharmaceuticals Limited, Fernbank House, Cheshire, UK) or Lid-Care® (Novartis International AG, Basel, Switzerland) cleaning solutions. Non pharmacological treatment/procedures included tear retention methods such as punctum plug, punctum cautery and tarsorrhaphy. (Table 1) Each recorded entry was considered as one patient episode (PE). In order to calculate the patient episodes, repeated visits by the same patient was identified by sorting the entries by patients ID and deleting the duplicates.

**Table 1:** The unit costs of dry eye treatments and their categories.

Treatment category	Unit Cost (US$)
**Meibomian gland disease therapy**	
Lid-Care*	7.76
Blephagel*	10.7
Eye mask (hot/cold)	5.35
**Preservative free lubricant eye drops**	
Refresh Plus eye drop‡	15.16
Tears Naturale Free‡	14.90
Refresh Ophthalmic Solution‡	8.82
**Preserved lubricant eye drops**	
Hypromellose 0.3% eye drop*	1.52
Tears Naturale II*	5.00
Refresh Tears*	6.65
Sodium Hyaluronate 0.1% (Hialid)*	8.88
Sodium Chloride 0.9% eye drop*	1.52
Fresh Tears*	5.35
Vidisept Ophtiole eye drop*	7.13
Hypo Tears eye drop*	3.30
Systane*	9.81
Liquifilm Tears*	5.88
**Lubricant Ointments and gels**	
Vidisic eye gel ¶	11.60
Duratears eye ointment ¶	5.88
Solcoseryl 20% eye gel ¶	5.35
Refresh Liquigel¶	8.92
**Cyclosporin eye drops**	
Restasis 0.05% ophthalmic emulsion‡	64.92
**Oral Supplements**	
Thera Tears capsule*	43.69
**Non Phamacological Treatments/Procedures**	
Punctum Cautery†	22.29
Punctum plug†	78.47
Tarsorrhaphy†	66.86

* Per bottle.

‡ Per 30 vials.

† Per procedure.

¶ Per tube.

Comparisons in each group between two years were carried out using Pearson Chi-Square test. Significance level was set at alpha  = 0.05.

## Results

**Figure 1:**
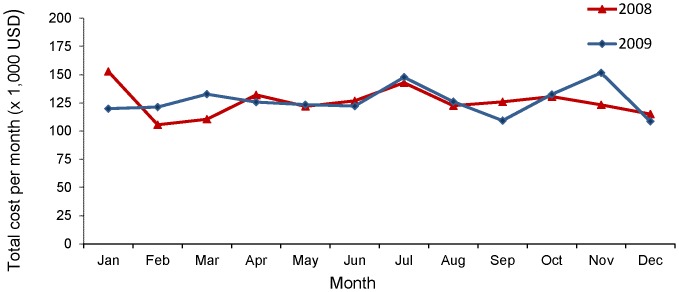
Distribution of total monthly expenditure on dry eye treatment in 2008 and 2009.

Cost data from 54,052 patients were available for analysis, from 2008–2009. Over this period (2008–2009), the total number of recorded patients’ episodes was 132,758. **Figure 1** shows monthly distribution of total expenditure on dry eye related medications and procedures in 2008 and 2009. Total annual expenditure on dry eye treatment for year 2008 and 2009 were US$1,509,372.20 and US$1,520,797.80 respectively and total expenditure per patient episode in year 2008 and 2009 were US$22.11 and US$23.59 respectively. There was only 0.8% increase in total annual expenditure from 2008 to 2009. However, expenditure per patient episode had increased by 6.69% in year 2009, compared to 2008. This is despite the 5.56% decrease in the total number of patients’ episodes from 2008 (68,278 episodes) to 2009 (64,480 episodes).

**Figure 2:**
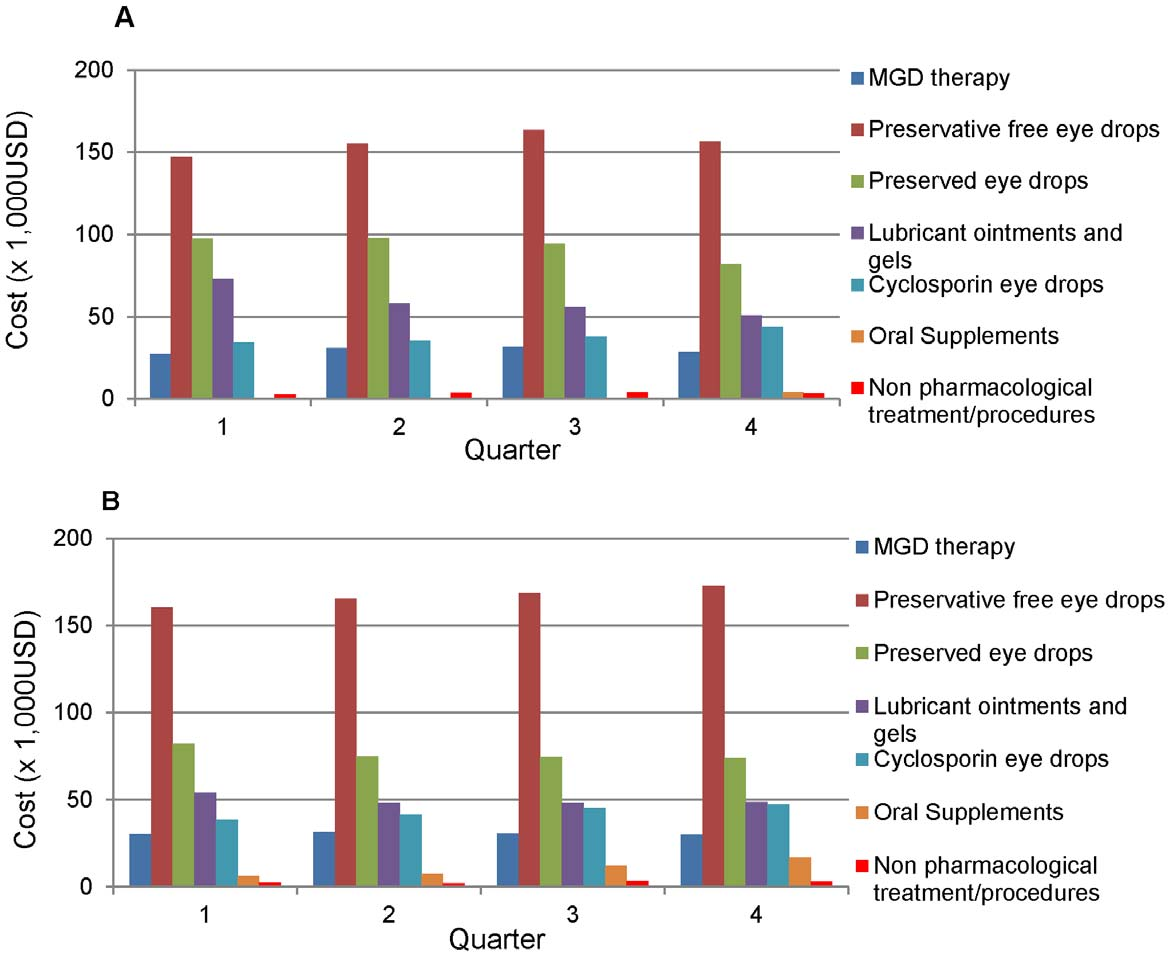
Distribution of quarterly expenditure among different treatment groups in 2008 & 2009. **Figure 2A:** Quarterly expenditure in 2008 **Figure 2B:** Quarterly expenditure in 2009.

The unit costs of different dry eye treatments and their categories are shown in Table 1. **Figure 2A and B** shows quarterly expenditure for different types of treatment in dry eye for 2008 and 2009 respectively. Oral supplements in the form of Thera Tears® (Advanced Vision Research, Inc., Woburn, MA, USA, Ciba-Geigy Corporation, Tarrytown, NY 10591 USA) were only available in pharmacy from last quarter of 2008. **Table 2** shows the Mean quarterly expenditure in 2008 (column 1) and 2009 (column 2) stratified by the type of dry eye treatment. The last column (**Table 2**) shows the expenditure per patient episode by type of treatment, in other words, we divided the total expenditure for each type of treatment by the number of episodes involving that treatment over 2008–2009.

Cost per patient episode for pharmacological therapies (**Table 2** **,** last column) such as Theratears® oral supplements and cyclosporine eye drops are relatively higher (US$80.27, US$143.09) compared to conventional therapies such as MGD therapy and lubricants (US$12.68, US$21.82). These forms of treatment however may not necessarily be utilized over the same duration of time. In order to examine the expenditure of same duration, one can compare the expenditure for these items per quarter (columns 1 and 2), which shows that the amount spent on lubricants are much higher compared to other types of treatments. Note that the unit cost of medications didn’t change over the period of the study (**Table 1**).

**Table 2:** Distribution of expenditure among different dry eye treatment groups.

	Quarterly Expenditure in 2008 (US$) (Mean±SD)	Quarterly Expenditure in 2009 (US$) (Mean±SD)	Expenditure per Patient Episode (US$)
**Phamacological Treatments**			
Meibomian gland disease therapy	29509.52±1936.56	30638.12±681.97	12.68
Preservative free lubricant eye drops	155461.90±6739.84	166837.49±5176.30	37.10
Preserved lubricant eye drops	92848.48±7311.95	76398.98±3837.25	12.45
Lubricant ointments and gels	59384.31±9477.15	49793.29±2769.97	19.96
Cyclosporin eye drops	37747.65±4262.05	43131.68±4060.93	143.09
Oral Supplements	4063.325†	10704.16±4828.84	80.27
**Non Phamacological Treatments/Procedures**			
Punctum Cautery	122.60±80.89	215.11±101.97	25.49
Punctum plug	2501.12±573.38	1634.14±655.65	87.98
Tarsorrhaphy	790.44±220.76	846.46±436.12	72.75

† Oral supplements were only available in pharmacy from last quarter of 2008.

**Figure 3:**
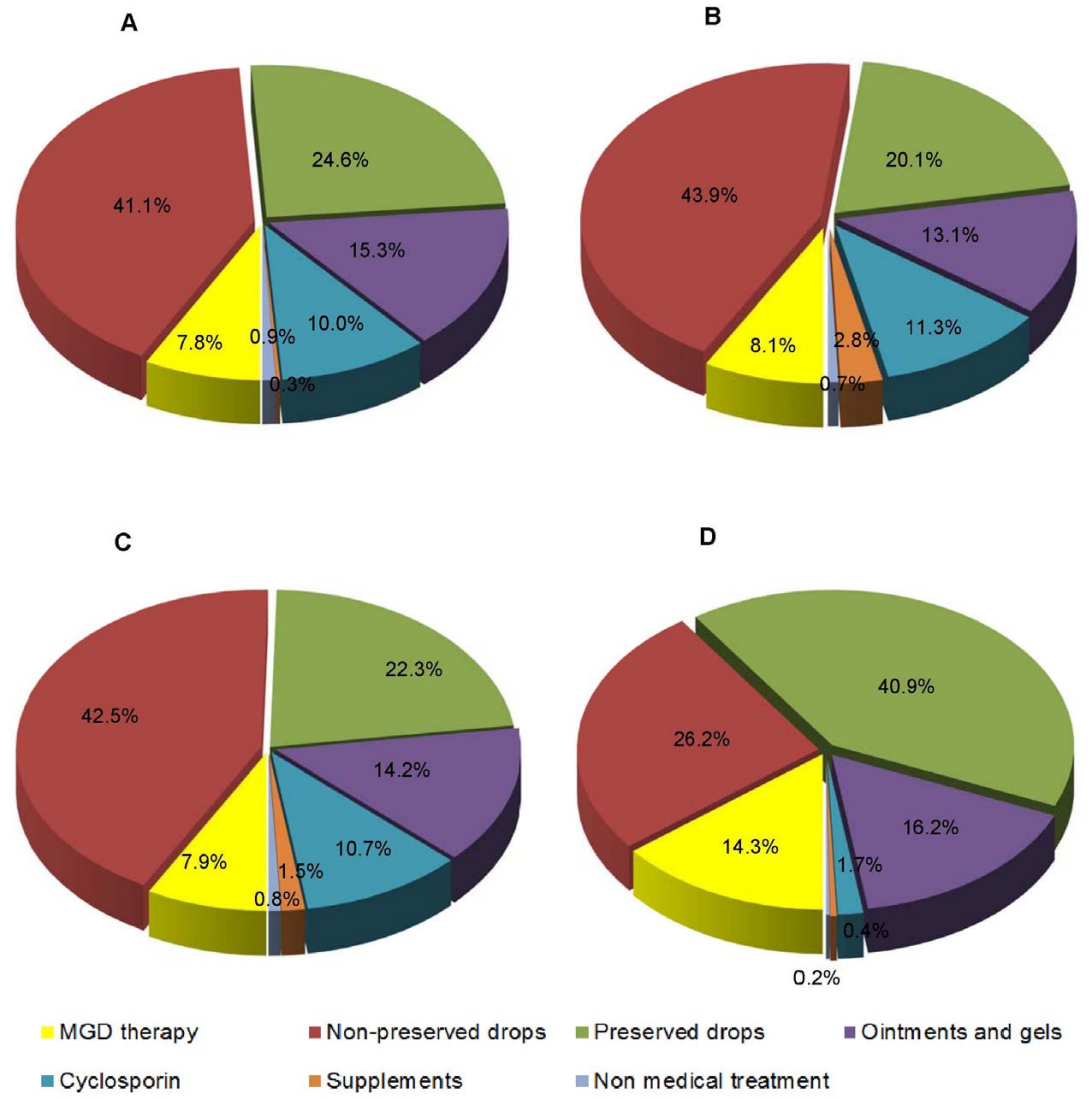
Relative expenditure & distribution of patient episodes of dry eye treatment categories in 2008 & 2009. **Figure 3A**. Relative expenditure in 2008. **Figure 3B** **.** Relative expenditure in 2009. **Figure 3C** **.** Total expenditure in 2008 & 2009. **Figure 3D:** Distribution of patient episodes in 2008 & 2009

The relative expenditure of dry eye treatment by category is shown in **Figure 3** **A** (for 2008), **B** (2009) and **C** (2008–2009 overall). As in **Figure 3C**, Pharmacological treatment attributes to 99.2% of the total expenditure and lubricants accounts for 79.3% of the total pharmacological treatment expenditure. Total number of units purchased in the categories of preservative free lubricants, 0.05% topical cyclosporine emulsion and MGD therapy have increased significantly (p<0.001) from 2008 to 2009 whereas the number of units purchased in preserved lubricants and ointments/gels have reduced significantly (p<0.001) (**Table 3**). The number of surgical procedures in general was not significantly different between 2008 and 2009(p = 0.085). As oral supplement was only available last quarter of 2008, this analysis was not possible for this type of treatment.

**Table 3:** Comparison of number of units purchased in each treatment group between 2008 and 2009.

	Total no of unitspurchased in 2008	Total no of unitspurchased in 2009	Comparison between 2008 and 2009 (p values)
**Phamacological Treatments**			
Meibomian gland disease therapy	13761	14469	<0.001*
Preservative free lubricant eye drops	42058	46905	<0.001*
Preserved lubricant eye drops	100763	90812	<0.001‡
Lubricant ointments and gels	26587	22755	<0.001‡
Cyclosporin eye drops	2326	2658	<0.001*
Oral Supplements	93	980	†
**Non Phamacological Treatments/Procedures**	366	316	0.085‡

* Increase in the number of units purchased.

‡ Decrease in the number of units purchased.

† Oral supplements were excluded from analysis as they were only available in pharmacy from last quarter of 2008.

## Discussion

Seven major classes of dry eye treatment modalities currently available in Singapore are, meibomian gland disease (MGD) treatment, preservative free lubricant eye drops, preserved lubricant eye drops, lubricant ointment and gels, anti-inflammatory medication such as cyclosporine eye drops and non pharmacological interventions such as punctum plugs, punctum cautery and tarsorrhapy.

In our study, total expenditure of dry eye treatment between 2008 and 2009 were US$0.06 million per 1,000 patients and pharmacological therapy was the major cost driver, accounting for 99.2% of total cost. In a previously published survey in US, on 2,171 respondents with dry eye which included both direct (cost of consultation and treatment) and indirect costs (productivity loss due to absenteeism and presenteeism) showed that average annual cost of managing a patient with dry eye in US was US$783 (variation, US$757 to US$809) from the payers’ perspective. When adjusted to the prevalence of dry eye nationwide, the overall burden of the disease for the U.S. health-care system would be US$3.84 billion and from a societal perspective, the average cost of managing dry eye was estimated to be US$11,302 per patient and US$55.4 billion to the U.S. society overall [10]. Another study that involved 6 European countries (France, Germany, Italy, Spain, Sweden and United Kingdom) showed that total annual healthcare cost of 1,000 DES sufferers managed by ophthalmologists ranged from US$0.27million in France to US$1.10million in UK [9]. These included the cost of specialist visits, diagnostic tests, pharmacological and non pharmacological treatment. Specialist visits were the primary cost driver in France, Germany and Spain where as diagnostics test were the primary cost driver in Italy and Sweden. In UK it was the prescription drugs. If we included the cost per consultations (ranged from US$108.00 to US$58.00 per visit), and diagnostic tests such as Schirmers (US$13.0 per test), meibography (US$40.65 per test) which is much higher compared to those European countries [9], and the indirect costs, annual DES related healthcare expenditure in our settings is likely to be much higher than estimated in this study.

It is also worth pointing out that Goods and Services Tax (GST) of 7% of the selling price in government subsidized patients has been absorbed by the institution. This would imply that the out of pocket actual expenditure on dry eye could have been greater, if patients were to purchase the same medications from a non-hospital pharmacy in Singapore. Between 2008 and 2009 there were a total of 58,469 subsidized patients’ visits (44% of total recorded patients’ episodes).

Our study suggests that lubricant eye drops are the mainstay of treatment for DES(67.1% of total PE) with a preference to preserved eye drops (40.9% of total PE) (Figure 3D). The lubricants with preservatives are much cheaper (mean unit cost US$5.50±2.81) compared to preservative free lubricants (mean unit cost US$12.96±3.59) and despite more episodes in this category (54,357) compared to preservative free lubricant eye drops (34,721), the overall expenditure is still lower in the preserved category (Figure 3C).

The unit cost of preservative-free lubricant eye drops tends to be more expensive than the preserved lubricant eye drops (Table 1). In order to evaluate whether the private patients who are probably financially better off than the subsidized patients, prefer the preservative free eye drops, we performed Chi-square test to evaluate the association of payment status of the patients with the preservative status of the lubricant eye drops purchased over the two years (2008–2009). Patients who did not qualify for the government subsidy were considered as private patients. We found that 53.3% of the pharmacy episodes of private patients were for preservative free medications, but this was only 21.3% among the subsidized patients (p<0.001). This shows that socioeconomic factors may play a role in determining the type of dry eye medications purchased by the patients. Previous studies have not compared the relative expenditure of preservative free versus preserved lubricants.

It is interesting to note that in our centre, the selling price of monthly supply of Cyclosporine eye drops were much lesser (US$64.92/patient) while cost of Thera Tears® oral supplement were quite higher (US$43.69/patient) compared to a previously published study in USA (US$115 and US$14 respectively) [2].

In our centre cost per one time procedure for punctum cautery, punctum occlusion and tarsorrhaphy were US$22.29, US$78.47 and US$66.86 per patient respectively, This is much less than in a previously published US study (US$163, US$305 and US$464/per patient respectively) [2].

The effects of punctum plugs, punctum cautery and tarsorrhaphy might be more permanent compared to lubricants and may be therefore more cost effective in the long term [11]–[13].

### Study Limitations

It is possible that we underestimated the burden of DES treatment because we excluded the data on cost of oral Doxycycline, topical Fucithalmic and Tetracycline ointments prescribed for meibomian gland disease and corticosteroids. We did not include these data because we couldn’t ascertain the exact diagnosis and indications for these treatments from our pharmacy database,

Lubricants may have been used to treat conditions other than dry eye. e.g. allergic conjunctivitis, recurrent corneal erosions and exposure keratopathy. However, we assumed that proportions of these conditions were probably low in our sample and would not skew the results of this study. Previous study on use of eye care and associated charges in US shows that case incidences of dry eye and blepharitis attributed to 75 to 78% of the total case incidences of external eye diseases [14].

Because of the unavailability of data, we didn’t include Non pharmacological therapies e.g. humidifiers and complementary and alternative medicine (CAM) therapies such as acupuncture and traditional Chinese medicine in our analysis. Previous study has shown amount spent on CAM dry eye products ranges from US$30 to US$2 per patient per month [15].

We didn’t estimate the cost of consultations which varies from US$108 to US$58 per visit, in our analysis as our database didn’t capture the exact diagnosis or purpose of a specific consultation visit to dry eye clinic. Our study showed lower annual expenditure compared to those studies that included physician consultation charges [9], [10], [15]. One study showed up to >70% of total direct costs were for physician consultations [15].

In our study, we didn’t consider the impact of severity of the DES on total expenditure and the different prevalence of each severity category. Previously published data showed that less severe DES is more common [9], [10]. DTS 2 and 1 are more common than DTS 3). In severe cases, combination therapy (eg. lubricants with punctum plugs and/or cyclosporine) is likely to be used and the actual expenditure of DES management in these more severe patients may have been higher than suggested in this paper. In US annual expenditure of DES by severity per patient: US$678 for mild DES, US$771 for moderate DES and US$1,267 for severe DES [10].

The data in this paper which only evaluated a hospital based pharmacy may underestimate the national burden of DES since some patients with dry eye may not come to hospital and either self-treat themselves with over the counter lubricating eye drops or are managed by general practitioners. Out of hospital pharmacies in Singapore may also be more accessible to the general public in terms of number of branches and locations. In previous cohort studies, that only 11% of dry eye sufferers seek professional help. Patients who self-diagnosed with dry eye, 57% purchased over the counter drops [2], [16]. Because of the recent economic downturn, purchases of over the counter lubricants without visiting doctor’s office might have increased over the period. To estimate the true impact of recent economic downturn on cost and patterns of DES expenditure, a larger population based study over several years is required.

Previously published survey on 74 patients has shown an estimated annual productivity loss of > US$5,000 per patient due to dry eye. [17] In our study, we didn’t take indirect costs and intangible costs such cost of travelling to doctor’s office for consultation, cost loss of productivity and time into consideration due to uncertainty of the accuracy of the data. Therefore total societal costs borne by the patient is likely to be much higher.

### Clinical Significance

Up to our knowledge there are no other studies published on the cost of DES management in Asia. Our study demonstrate that DES impose a direct burden to the health care expenditure and given the limitations of the availability of socio-economic data in our data sources, true costs of DES, borne by both the patient and the government, are likely to be much higher. Outcome of this study can be used in conjunction with clinical trials, and quality of life studies to determine the cost effectiveness of the dry eye treatment. It will also contribute to increase the awareness of clinicians and policymakers on the importance of pursuing cheaper and effective novel treatment modalities and improving the existing public healthcare systems to reduce the financial burden of DES on both patients and healthcare systems. Our data seems to show lower expenditure compared to studies that included physician consultation charges [15], suggesting that national health care costs may be reduced if stable patients could be managed by primary health care practitioners such as general practitioners, optometrists or even self medicated.

In summary, DES seems to impose a substantial economic burden to the health care expenditure in Singapore. In our centre, mean costs of dry eye treatment per year is estimated to be US$1,515,085.00±8,079.12. Lubricants accounts for a large proportion of the pharmacological expenditure and number of units purchased on certain categories of treatment such as MGD therapy, preservative free lubricants and cyclosporine eye drops were shown to increase significantly over the period(P<0.01). Given the limitations of the availability of socio-economic data in our sources, true societal costs of DES are likely to be much higher than estimated.
